# Research on the aging characteristics of base liquor in Sichuan Province’s strong Aroma Baijiu at different aging periods using mass spectrometry and 3D fluorescence spectroscopy methods

**DOI:** 10.1371/journal.pone.0344656

**Published:** 2026-04-30

**Authors:** Qing He, Guoquan Han, Hongfei Yao, Jun Wang, Dongxian Geng, Qiuyue Han, Xiao Wang, Kechun Tang

**Affiliations:** 1 Sichuan Agricultural University, Ya’an, Sichuan, China; 2 Sichuan Provincial Institute of Product Quality Supervision, Inspection and Testing, Chengdu, Sichuan, China; 3 Sichuan Industrial Metrology and Testing Institute, Chengdu, Sichuan, China; 4 Sichuan Provincial analysis and Testing Center, Chengdu, Sichuan, China; 5 Sichuan Alcohol Research Institute Co Ltd, Chengdu, Sichuan, China; 6 Chengdu Analytical Applications Center, Shimadzu (China) Co Ltd, Chengdu, Sichuan, China; University of Kelaniya, SRI LANKA

## Abstract

The aging process of Baijiu directly impacts its flavor and quality, yet the chemical changes during aging lack systematic analysis. This study focused on Luzhou-flavor base liquor from Sichuan Province, aiming to elucidate the compositional evolution across different aging periods and provide a scientific foundation for Baijiu quality enhancement. Volatile flavor compounds were detected using gas chromatography–mass spectrometry (GC-MS), and metabolomic analysis was employed to screen multiple potential biomarkers. Combined with OPLS-DA, their variation characteristics across different aging periods were further investigated. In addition, three-dimensional fluorescence spectroscopy was employed to investigate the correlation between flavor components and aging duration, while odor activity values (OAVs) were calculated to evaluate the contribution of key compounds to the overall flavor profile. Results showed that 75 flavor compounds were detected across five aging periods (1, 5, 10, 15, and 20 years). As aging progressed, the concentration of esters decreased significantly from 95.56 mg/L at year 5 to 24.04 mg/L at year 20. Acid content peaked at 97.29 mg/L in the fifth year and gradually declined thereafter. Alcohols and polyphenols reached their maximum concentrations at year 10 (87.96 mg/L) and year 15 (80.67 mg/L), respectively, followed by a marked decline. These trends reveal the dynamic evolution of different flavor substances throughout the aging process. Among these changes, a “redshift” phenomenon in fluorescence spectra was observed, reflecting increased molecular complexity and a notable enhancement in flavor richness. These findings highlight the critical impact of the chemical evolution of base liquor components on aroma and quality. The results provide theoretical support for understanding the aging mechanism of Baijiu and optimizing its processing techniques, laying a scientific foundation for quality control and market promotion efforts.

## 1. Introduction

With the development of modern food industries and growing consumer demand for healthier beverages, the quality assurance and flavor optimization of traditional fermented foods have received increasing attention. As a beverage with a long history of brewing, the flavor characteristics of Chinese Baijiu are primarily influenced by factors such as brewing techniques, storage conditions, and aging duration. During the aging process, the chemical components of the base liquor undergo dynamic changes, directly affecting its sensory attributes, including color, aroma, and taste [1]. However, the chemical mechanisms underlying the Baijiu aging process remain insufficiently understood, hindering scientific optimization of Baijiu quality and effective control of the aging process.

Current research on Baijiu quality and flavor characteristics mainly focuses on key substances such as volatile compounds, acids, and esters. Mu et al. [2] and Zheng et al. [3] used mass spectrometry to quantitatively analyze ester compounds in aged Baijiu and found that changes in ester content over time are crucial to aroma formation. Zhang et al. [4] applied three-dimensional fluorescence spectroscopy to study the fluorescence variations of polyphenols and phenols in Baijiu, indicating that fluorescence characteristics can indirectly reflect the increasing molecular complexity during aging. However, existing studies often rely on single analytical methods, failing to comprehensively reveal the dynamic changes of Baijiu’s multi-component system during aging. Additionally, the interactions among chemical components at different aging stages and their overall impact on flavor lack systematic analysis, limiting a deeper understanding of Baijiu aging mechanisms.

To fill these gaps, this study combined mass spectrometry with three-dimensional fluorescence spectroscopy to comprehensively analyze the chemical composition of Luzhou-flavor Baijiu base liquors across different aging periods. Two complementary strategies were employed in the application of mass spectrometry: headspace solid-phase microextraction coupled with gas chromatography–mass spectrometry (SPME-GC-MS) was used to efficiently capture and resolve volatile flavor compounds in Baijiu samples; in parallel, derivatized liquid samples were subjected to GC-MS-based metabolomic analysis to identify potential biomarkers and complex metabolic features. The former approach focused on the dynamic evolution of key flavor substances, while the latter expanded the overall understanding of the chemical composition of Baijiu base liquors. When integrated with three-dimensional fluorescence spectroscopy, these methods not only enabled precise detection of trace components but also revealed fluorescence characteristic changes during the aging process, thereby systematically elucidating the chemical evolution patterns of Baijiu base liquors over time. The study found significant changes in the concentration and distribution of key components such as esters, acids, and polyphenols across different aging stages. Furthermore, a “redshift” phenomenon was observed in the fluorescence spectra as aging progressed, indicating increasing molecular complexity and enhanced flavor richness in Baijiu base liquors. This study not only uncovers the dynamic evolution patterns of different chemical components during aging but also clarifies the pathways through which key components influence Baijiu flavor and quality, enriching the theoretical framework of the aging process. By revealing the dynamic evolution of Baijiu base liquor components from multiple perspectives, the research provides scientific support for understanding Baijiu aging mechanisms, improving quality, and optimizing processes.

## 2. Materials and methods

### 2.1. Materials and reagents

In studies distinguishing Baijiu based on aging periods, the primary focus was on developing a differentiation model [5]. The materials and tools used in this study included Baijiu samples obtained from a renowned Sichuan Baijiu brand, encompassing various aging stages: 1, 5, 10, 15 and 20 years. These samples represent different aging periods, enabling a comprehensive analysis of the effects of aging on Baijiu characteristics [6]. In addition, to simulate the blending process of base liquors in actual Baijiu production, three groups of mixed samples were designed to represent blended liquors with weighted average aging periods of 5, 10, and 15 years. Specifically, the 5-year sample was prepared by mixing 70% of 1-year-aged base liquor with 30% of 10-year-aged base liquor; the 10-year sample was obtained by blending 5-year and 10-year base liquors in equal proportions (50:50); and the 15-year sample was formulated using 70% of 20-year-aged base liquor and 30% of 5-year-aged base liquor.

To ensure that the equivalent aging years of the blended samples possess clear physical and chemical significance, this study introduces an exponential effective aging degree model based on the kinetic characteristics of the Baijiu aging process. During Baijiu aging, multiple physicochemical processes—including esterification, oxidation, volatilization, and molecular association—occur simultaneously. The overall flavor evolution therefore manifests as a time-dependent process that gradually approaches stability. Previous studies have demonstrated that such complex aging behavior can be regarded as a comprehensive kinetic effect resulting from the superposition of multiple reactions, and is thus suitable for approximation using effective kinetic models [7]. In food quality evolution studies, first-order or pseudo-first-order kinetic models are widely employed to describe the monotonic temporal evolution of quality or state indicators [8]. Based on these considerations, the overall aging degree of the liquor body is defined in this study as a normalized indicator that increases monotonically with aging time and gradually approaches a stable state. Its mathematical expression is given as follows:


$$M\left(t\right)=1-e^{-kt}$$ (1)

where k represents the maturation rate constant, reflecting the overall reaction rate during the aging process. This parameter does not represent the rate constant of any single chemical reaction; rather, it reflects an effective kinetic scale characterizing the overall aging progression. Its value is determined with reference to reported kinetic characteristics of key reactions involved in Baijiu aging—such as esterification—based on previous studies [9].

The equivalent aging time T of a blended liquor sample is defined as the aging duration of a single-year base liquor that exhibits the same overall aging degree as the blended system. Assuming that the overall aging state of the blended liquor can be expressed as the volume-fraction–weighted sum of the aging degrees of its individual components, the comprehensive aging degree of the blended sample can be denoted as $\sum_{i}w_{i}M\left(t_{i}\right)$. By substituting this value into Equation (1) and inversely solving for time, the expression for the equivalent aging time of the blended liquor sample can be obtained as follows:


$$T=-\frac{\text{1}}{k}ln\;\left(\sum_{i}w_{i}e^{-kt_{i}}\right)$$ (2)

In the equation, wᵢ denotes the volume fraction of each component, and tᵢ represents the corresponding actual aging time. The definition of the equivalent aging time proposed above is intended to characterize the overall aging state of blended liquors from a kinetic perspective, ensuring consistency and reproducibility in the classification of aging degrees among different blended samples. It is not designed to provide an exact representation of the actual storage duration or the sensory aging perception of Baijiu.

Internal standards were used to ensure accuracy, including a mixed reference solution of 4-bromofluorobenzene and 1,2-dichlorobenzene-D4 provided by O2SI (USA), as well as a reference solution of acenaphthene-D10 at a concentration of 100 μg/L [10]. To ensure the comparability of samples and the validity of experimental data, all Baijiu base liquor samples were derived from the same fermentation batch and produced under identical technological conditions. Following packaging, the samples were aged separately in a standardized storage facility under uniform conditions—constant temperature (18 ± 1 °C), relative humidity (70 ± 5%), and protection from light and air exposure. Throughout the storage period, all samples remained sealed and undisturbed, ensuring that the only variable across different aging groups was storage duration. This rigorous control effectively eliminated potential confounding factors and ensured the reliability of the comparative analysis.

### 2.2. Instruments and equipment

In this study, the detection of volatile flavor compounds and the metabolomic analyses were both conducted using a GCMS-TQ8050 NX triple quadrupole gas chromatography–mass spectrometry system (Shimadzu, Japan). This instrument was employed for the separation and qualitative/quantitative analysis of trace volatile organic compounds in Baijiu base liquor samples. The compound identification procedures were consistent across both types of analyses, relying on retention times and mass spectral fragmentation patterns, and confirmed through comparison with reference spectra in the Shimadzu Intelligent Aroma Database.

Automated sample injection was performed using the PAL AOOC-6000 autosampler, equipped with a heated magnetic stirring module to stabilize sample handling conditions and ensure consistent injection. Volatile compounds were extracted using the PAL SPME Arrow solid-phase microextraction system, which utilized coated fibers to enrich analytes prior to direct introduction into the GC-MS system for analysis.

Three-dimensional fluorescence spectroscopy was conducted using a HORIBA Aqualog fluorescence spectrophotometer (HORIBA, Japan). The instrument employs a xenon lamp as the excitation source and supports wide-range wavelength scanning, making it suitable for detecting fluorescent compounds in complex samples. The fluorescence data were used to assess changes in luminescence characteristics among samples from different aging periods.

### 2.3. Processing methods

All Baijiu base liquor samples in this study, including both the original aged samples and the three blended samples with weighted average aging periods (5, 10, and 15 years), were subjected to metabolomic analysis following the same standardized procedure. For the analysis of volatile flavor compounds in Baijiu base liquors, sample preparation was conducted using a PAL SPME Arrow solid-phase microextraction device. Specifically, 10 mL of Baijiu sample was transferred into a 20 mL headspace vial, and an SPME Arrow fiber was inserted. The sample was equilibrated and adsorbed at 55 °C for 30 minutes. The fiber was then directly introduced into the GC-MS injection port for the enrichment and detection of trace volatile components in the base liquor.

To prepare Baijiu base liquors samples for metabolomics analysis, the following procedure was adopted. First, 200 μL of the sample was mixed with 50 μL of methoxyamine pyridine solution (5 mg/mL). The mixture underwent an oxidation reaction at 70°C for 30 minutes. This reaction stabilizes analytes by converting carbonyl compounds into oximes, improving detection rates in subsequent analyses [11].

After the oxidation step, 50 μL of MSTFA (N-methyl-N-(trimethylsilyl)trifluoroacetamide) was added. MSTFA serves as a derivatization reagent, converting hydroxyl and other functional groups into trimethylsilyl derivatives, enhancing analyte volatility and stability for GC-MS analysis [12]. The reaction with MSTFA continued for 30 minutes. After derivatization, the supernatant containing the derivatized analytes was centrifuged and used for GC-MS analysis.

After derivatization and centrifugation of the samples, quantification of the target compounds was performed using the internal standard method. Acenaphthene-D10 (100 μg/L) was used as the internal standard. The concentration of each analyte was estimated by calculating the peak area ratio between the target compound and the internal standard, and applying the following equation:


$$C=\left(\frac{A_{\text{target}}}{A_{\text{internal}}}\right)\times C_{\text{internal}}$$ (3)

In the equation, C represents the concentration of the target compound (mg/L), $A_{\text{target}}$ denotes the peak area of the target compound, $A_{\text{internal}}$ is the peak area of the internal standard, and $A_{\text{internal}}$ is the concentration of the internal standard.

Prior to three-dimensional fluorescence spectroscopy analysis, all Baijiu base liquor samples were filtered through a 0.22 μm microporous membrane to remove suspended particles and prevent fluorescence scattering interference. During measurement, the excitation wavelength range was set from 220 to 500 nm with a step size of 5 nm, and the emission wavelength range was set from 250 to 600 nm with a step size of 2 nm. The scanning speed was 1200 nm/min, and both the excitation and emission slit widths were set at 5 nm. Each sample was measured in triplicate, and the average value was used. All fluorescence intensity data were subjected to background subtraction. The resulting data were processed using Origin and TBtools software to generate three-dimensional contour plots. The main fluorescence peak positions (Ex/Em) and corresponding intensity values were extracted for characteristic analysis.

For metabolomics analysis, gas chromatography-mass spectrometry (GC-MS) was the primary technique. The initial column temperature was set at 50°C and maintained for 2 minutes to allow equilibrium [13]. The temperature was then ramped at 5°C/min to 130°C, followed by a slower increase at 2.5°C/min to 160°C. Finally, the temperature was increased at 5°C/min to a maximum of 300°C, which was held for 10 minutes to ensure complete elution of compounds. Each Baijiu base liquor sample was independently measured three times during GC-MS analysis to minimize experimental error and enhance data reliability. The final results were expressed as the average of the three replicate measurements and used for subsequent concentration calculations and statistical analysis.

The injection volume was 1 μL, with a splitless mode and helium as the carrier gas at a flow rate of 1 mL/min. The injector temperature was set to 290°C. The mass spectrometer operated in electron ionization (EI) mode with an ionization energy of 70 eV, producing ion fragments for compound identification. The mass spectrometer scan range was 50–550 m/z (mass-to-charge ratio), allowing detection and identification of a broad range of ions. The ion source temperature was maintained at 190°C to optimize ionization and detection.

### 2.4. Data processing

To ensure the accuracy and reproducibility of the research results, multiple statistical and visualization methods were employed to process and present the experimental data. First, TBtools and Origin software were used to visualize the flavor compounds of Baijiu base liquor samples across different aging periods. Heatmaps were generated to intuitively display the concentration trends of key flavor components, with color intensities reflecting variations among variables [14]. For multivariate discriminant analysis, Orthogonal Partial Least Squares Discriminant Analysis (OPLS-DA) was performed using SIMCA software to distinguish chemical composition differences among samples from different aging periods. By removing orthogonal components unrelated to sample classification, the model’s discrimination accuracy and explanatory power were enhanced. To validate the robustness of the OPLS-DA model, 200 permutation tests were conducted to prevent overfitting and ensure statistical significance. Variable Importance in Projection (VIP) scores were calculated to identify key compounds with significant contributions to sample classification. Variables with VIP scores greater than 1 were considered significant and regarded as potential differential markers. The data processing and visualization techniques employed in this section provided the technical foundation for the subsequent result figures and statistical analyses, ensuring a scientifically sound and statistically supported presentation of flavor evolution characteristics across different Baijiu aging periods [15].

## 3. Results and analysis

### 3.1. Comparison of flavor components and relative contents across aging periods

Compound Discoverer 2.0 software was used to analyze differences in peak area ratios of compounds in Baijiu base liquor samples from different aging periods. This analysis identified 49 representative differential compounds (see Table 1) whose concentrations varied significantly across aging stages. These compounds can serve as potential biomarkers to reflect the aromatic characteristics and chemical composition changes of Baijiu base liquors during the aging process. In Table 1, the listed peak areas represent the average values of each compound across the five aging periods. To ensure the scientific validity and comparability of the odor descriptions, the sensory characteristics of each compound were primarily referenced from the Shimadzu Smart Aroma Database, supplemented by known olfactory properties of the respective compounds. It should be noted that some compounds in Table 1 had similarity scores below 90. To maintain analytical rigor, a conservative interpretation strategy was applied when incorporating these compounds into subsequent statistical analyses. These compounds were mainly used to illustrate concentration trends and sensory contribution tendencies, but were not treated as primary biomarkers for conclusive interpretations.

**Table 1:** List of markers.

Name of odor compound	Similarity	Smell Description	Retention time
Ethyl formate	80	pungent	1.734
Ethyl acetate	84	pineapple	1.907
1,1-diethoxyethane	86	fruit,cream	2.042
Ethyl propanoate	85	fruit	2.49
Diacetyl	92	butter	2.639
2-Pentanone	90	ether,fruit	2.639
Propyl acetate	96	solvent,sweet, perfumed	2.625
1,1-Diethoxy-2-methylpropane	95	fruit	2.721
Ethyl butanoate	98	apple	3.381
Ethyl 3-methylbutanoate	86	fruit	3.91
Butyl acetate	89	pear	3.957
Ethyl valerate	98	yeast,fruit	5.634
1-Butanol	98	medicine,fruit	5.753
Pentyl acetate	94	banana	6.486
Methyl hexanoate	97	fruit,fresh,sweet	6.789
Ethyl isocaproate	83	fruit	7.011
Isoamyl alcohol	97	whiskey,malt,burnt	7.295
Ethyl hexanoate	80	apple peel,fruit	8.037
1-Pentanol	98	balsamic	8.214
Isoamyl butyrate	91	fruit	8.634
Hexyl acetate	98	fruit,herb	8.743
Propyl hexanoate	98	fruit	9.682
Ethyl heptanoate	77	fruit	9.96
2-Nonanone	91	hot milk,soap	10.841
Butyl hexanoate	96	fruit	11.346
Ethyl octanoate	97	fruit,fat	11.689
Acetic acid	97	sour	11.667
Furfural	97	bread,almond,sweet	11.829
Isopentyl hexanoate	98	fatty acids,fruity	12.095
Benzaldehyde	99	almond,burnt sugar	12.737
Furfuryl Acetate	92	fruit	13.025
Ethyl nonanoate	84	fruity,rose,nutty	13.223
Ethyl DL-Leucate	97	sweat	13.206
1-Octanol	92	chemical,metal,burnt	13.429
Isobutyric acid	91	rancid,butter,cheese	13.448
Hexyl hexanoate	97	apple peel,peach	14.271
Ethyl decanoate	97	grape	14.631
Isovaleric acid	92	sweat,acid,rancid	14.821
Diethyl succinate	96	wine,fruit	14.974
Valeric acid	96	sweat	15.662
Naphthalene	97	tar	15.694
Ethyl phenylacetate	99	fruit,sweet	16.312
2-Phenylethyl acetate	96	rose,honey,tobacco	16.667
Ethyl 3-phenylpropionate	95	flower	17.499
2-Phenylethanol	96	honey,spice,rose, lilac	17.689
Butylated hydroxytoluene	95	musty	17.864
Phenol	90	phenol	18.662
Ethyl myristate	92	ether	19.463
Ethyl palmitate	96	wax	21.561

### 3.2. Clustering analysis of flavor components across aging periods

Gas chromatography–mass spectrometry (GC-MS) was employed to systematically analyze the volatile trace compounds in Baijiu base liquor samples [16,17], resulting in the identification of 75 flavor-related compounds, as detailed in Table 2. These compounds represent the main compositional characteristics of Baijiu base liquors at different aging stages [18,19]. However, not all compounds were detected across all five aging periods, indicating clear temporal specificity and dynamic variability in their distribution. Such differences reflect the complex chemical evolution of Baijiu during maturation and reveal the distinct roles that various substances play in shaping the overall flavor at different stages. Considering the varying contributions of different compound classes to flavor formation, six representative categories—esters, alcohols, acids, phenols, aldehydes, and ketones—were selected as the focus of further analysis [20,21].

**Table 2:** Categories of Flavor Compounds in Baijiu Base Liquors and Their Flavor Contributions.

Compound Category	Number	Contribution to Flavor and Aroma (Summary)
Esters	40	The predominant contributors to Baijiu aroma, generally imparting fruity, floral, and sweet notes. Their dynamic changes during aging significantly influence the harmony, softness, and layered complexity of the liquor.
Aldehydes	7	Low-threshold compounds with strong sensory impact. They contribute fresh, nutty, or roasted notes, and also act as important precursors in reactions with acids and alcohols during aging.
Alcohols	7	Present in relatively high amounts, enhancing body fullness and texture. In small quantities, they enrich flavor complexity, while excessive levels may introduce pungency. They also serve as precursors for ester formation.
Acids	9	Provide sourness and balance, and are essential precursors for esterification. Their concentration changes directly affect the smoothness and coordination of the liquor’s flavor profile.
Ketones	4	Usually with relatively high odor thresholds, making limited direct contributions to aroma. However, they provide supportive fruity, fatty, or creamy nuances and enhance complexity in the middle and later stages of flavor development.
Phenols	3	Contribute medicinal, woody, and smoky characteristics. Despite their relatively low concentrations, they play a key role in shaping Baijiu’s individuality and aging traits, with some also exhibiting antioxidant properties.
Other compounds	5	Including amino acids, terpenes, sulfur compounds, aromatic hydrocarbons, and furans. Although less abundant, they often have unique or low-threshold aromas that enrich the subtlety and depth of the overall flavor profile.

**Fig 1:**
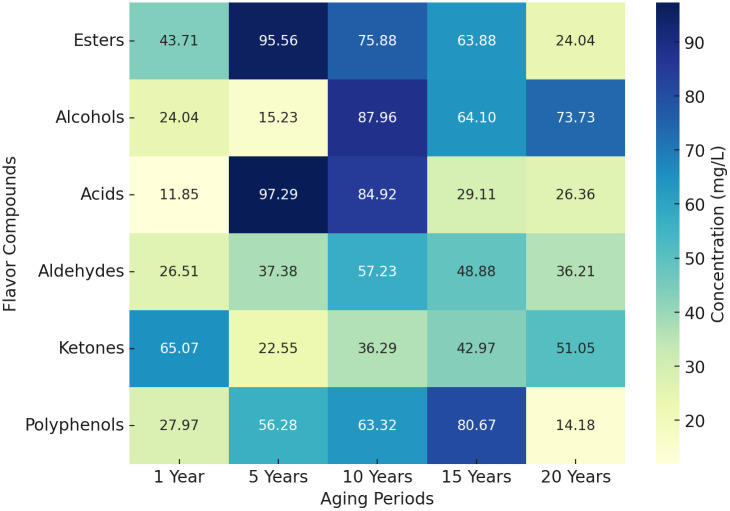
Heatmap Analysis of Flavor Compound Concentrations Across Different Aging Periods.

To reveal the dynamic evolution of flavor substances at different aging stages, a heatmap (Fig 1) was generated to visualize the relative concentration changes of six key compound classes (esters, alcohols, acids, aldehydes, ketones, and polyphenols) across all aging periods. Fig 1 show that ester compounds reached their highest average concentration (95.56 mg/L) in the 5-year-aged samples, followed by a gradual decline to 24.04 mg/L at 20 years, indicating a decreasing trend likely due to hydrolysis or oxidative degradation during the aging process. Alcohol compounds exhibited the highest concentration (87.96 mg/L) in the 10-year sample, with values ranging from 15.23 to 73.73 mg/L in other periods, suggesting a pattern of accumulation and transformation over time. Acid compounds showed a marked increase in concentration at 5 years (97.29 mg/L), followed by a decrease to 84.92 mg/L at 10 years and a further downward trend thereafter. This pattern suggests that acids peak during mid-term aging, likely associated with the hydrolysis of esters. Aldehyde compounds displayed a fluctuating upward trend, reaching a peak concentration of 57.23 mg/L at 10 years. Although levels declined thereafter, they remained relatively high, indicating that aldehydes continue to contribute to flavor in the later stages of aging. Ketone compounds were already present at relatively high concentrations in the 1-year-aged sample (65.07 mg/L), showed a slight decrease during mid-aging, and rose again to 51.05 mg/L at 20 years, indicating their stability and sustained role throughout the entire aging period. Polyphenolic compounds exhibited a trend of initial increase followed by a decline across different aging stages: the concentration was 27.97 mg/L at 1 year, gradually rising to a peak of 80.67 mg/L at 15 years, and then markedly decreasing to 14.18 mg/L at 20 years. This pattern indicates that polyphenols play a prominent role during mid-term aging, while their reduction in later stages may result from polymerization reactions or sedimentation.

### 3.3. OPLS-DA and OAV analysis of flavor components across aging periods

Given that the 1-, 10-, and 20-year-aged base liquor samples represent the key stages of early, mid-, and long-term storage, respectively, they effectively illustrate the dynamic changes in chemical composition during the aging process. Therefore, these three aging periods were selected for orthogonal partial least squares discriminant analysis (OPLS-DA) and odor activity value (OAV) analysis to highlight the critical stages of flavor evolution.

**Fig 2:**
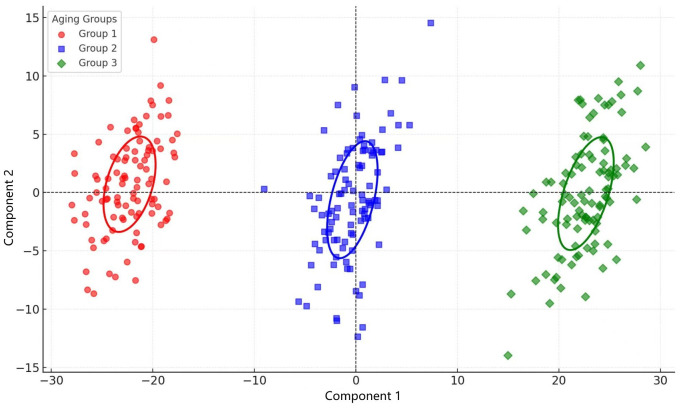
Score plot of OPLS-DA for different aging periods (1 year, 10 years, 20 years) of Baijiu base liquors.

Fig 2 presents the score plot derived from the OPLS-DA model analysis, where samples from different aging periods show distinct separations in the principal component space. Component 1 primarily reflects differences in the concentrations of ester compounds (e.g., ethyl acetate and ethyl hexanoate), while Component 2 correlates with acid compounds (e.g., hexanoic acid). The distribution indicates that 1-year-aged samples are mainly concentrated in the negative region of Component 1, with a smaller range along Component 2. This suggests that 1-year samples have relatively higher ester content and lower acid concentrations, resulting in a fresh and fruity flavor profile. In contrast, 10-year-aged samples shift significantly towards the positive direction of Component 1 and show an expanded range along Component 2. This indicates that mid-term aging involves partial transformation of esters and accumulation of acids (e.g., hexanoic acid), leading to a more balanced flavor profile with moderated acidity. By the 20th year, the samples are distributed further into the positive region of Component 1, with an even greater range along Component 2. This reflects the ongoing generation and transformation of acids during long-term aging, contributing to a more complex and full-bodied flavor profile. Additionally, the confidence ellipses for different aging groups reveal variations between groups and the cohesion within each group. The smaller ellipse for the 1-year samples indicates higher consistency in chemical composition, while the expanding ellipses for the 10-year and 20-year samples highlight the increasing diversity and complexity of chemical reactions during mid- and long-term aging. These patterns align closely with the gradual degradation of esters, the accumulation of acids, and the dynamic changes in other compounds.

To quantify the dominant contributions of different compounds to the overall aroma, this study introduced the Odor Activity Value (OAV) as a supplementary analytical indicator. OAV is defined as the ratio of a compound’s actual concentration in the sample to its olfactory threshold; the higher the OAV, the greater the compound’s sensory significance in the overall aroma profile. Although a total of 75 volatile compounds were detected, only 49 key compounds were ultimately selected for OAV calculation. The results showed that ethyl hexanoate (OAV = 154.2), ethyl lactate (OAV = 93.4), acetic acid (OAV = 81.6), and ethyl acetate (OAV = 75.8) exhibited the highest OAVs, indicating their dominant contribution to the overall aroma intensity. In contrast, hexanoic acid (OAV = 18.2) [22] and n-propanol (OAV = 12.5) had lower OAVs but displayed increasing trends with aging time. These results suggest that ester compounds dominate the fruity notes in early aging, while acid compounds progressively enhance aroma balance in mid-to-late stages.

**Fig 3:**
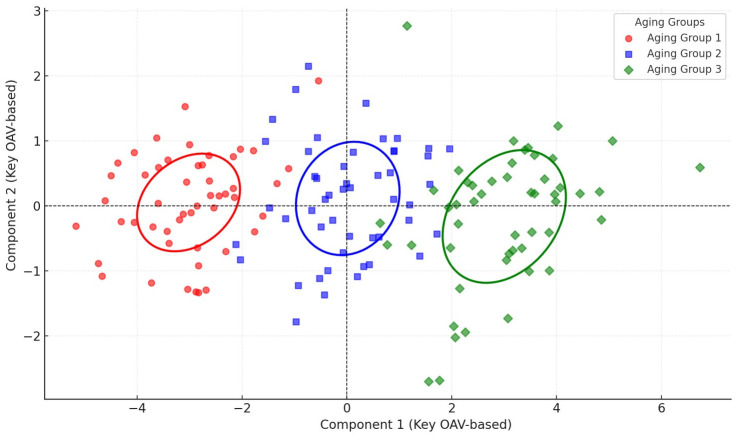
Odor activity value (OAV) contributions of key compounds for different aging periods (1 year, 10 years, 20 years) of Baijiu base liquors.

Fig 3 further validates the significant differences in chemical composition characteristics among samples from different aging periods, as depicted in Fig 2(a). Using OAV (odor activity value) analysis based on key compounds, the samples from 1, 10, and 20 years form distinct separations within the principal component space of odor contributions. The variation trends of Component 1 and Component 2 align closely with those observed in Fig 2. Specifically, Component 1 is primarily driven by ester compounds, while Component 2 reflects the contributions of acid compounds. From the perspective of odor activity values, the dynamic changes of these key compounds further enhance the grouping effects of samples from different aging periods in terms of flavor characteristics. This analysis also complements the OPLS-DA results by providing additional explanations of chemical composition differences from an odor contribution standpoint. These findings offer more precise evidence for understanding the influence of key compounds on overall flavor during the Baijiu base liquors aging process, deepening scientific insights into the interplay between chemical evolution and sensory attributes.

### 3.4. Three-dimensional fluorescence spectroscopy analysis of flavor components across aging periods

**Fig 4:**
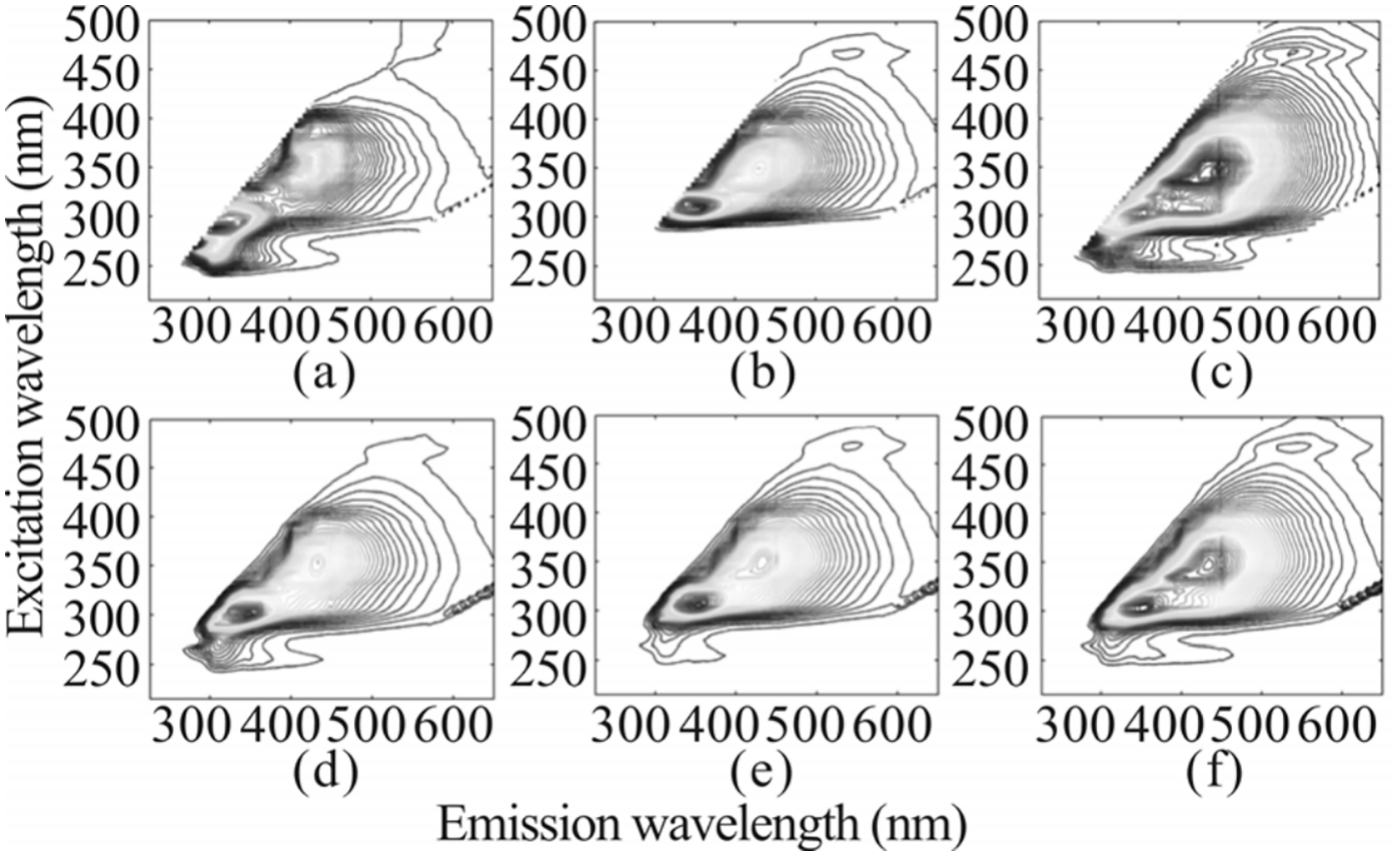
The 3D fluorescence spectra of the original base liquors with ages of (a) 1 years, (b) 5 years and (c) 10 years, as well as the blended aged liquors with weighted ages of (d) 5 years, (e) 10 years and (f) 15 years.

As shown in Fig 3, detailed three-dimensional fluorescence spectra of original base liquors and 37 blended aged Baijiu base liquors samples were collected and analyzed to generate numerous contour plots. Fig 4(a) to 4(c) depict contour plots of the original base liquors aged for 1, 5, and 10 years, respectively. Each plot illustrates the unique fluorescence characteristics corresponding to the specific aging period of the base liquor. Differences observed in these fluorescence spectra suggest the chemical and physical changes occurring during Baijiu base liquors aging. Changes in fluorescence intensity and peak positions reflect the evolving nature of compounds present in Baijiu base liquors over time.

In contrast, Fig 4(d) to 4(f) present the averaged contour plots for blended aged Baijiu base liquors with weighted ages of 5, 10, and 15 years. These averaged plots aggregate data from multiple samples, providing a general fluorescence profile for each aging category. By averaging the data, these plots offer a clearer depiction of the overall fluorescence characteristics associated with blended Baijiu base liquors at different aging stages, smoothing individual sample variations and highlighting broader trends.

Table 3 summarizes the fluorescence spectral parameters extracted from these contour plots. These parameters are critical for understanding the differences in fluorescence characteristics across aging periods. By analyzing these spectral features, Baijiu base liquors samples can be more finely differentiated based on their aging and blending characteristics. This detailed analysis enhances understanding of how aging and blending impact Baijiu base liquors’s fluorescence properties. Such information is essential for characterizing Baijiu base liquors and assessing its quality, as it reflects the impact of time and blending on its chemical traits.

**Table 3:** Fluorescence spectral characteristics of three base liquors and three blended aged liquors.

Liquor ages	Original ages			Weighted ages		
	1	5	10	5	10	15
Peak numbers	2	3	2	3	3	3
Optimal excitation wavelength (nm)	355	350	345	355	350	345
Peak wavelength (nm)	310 432	344 430 536	434 536	336 434 544	340 434 538	340 434 538
Peak intensity (×10^5^)	5.58	6.05	7.64	3.75	5.29	6.60

An analysis of the fluorescence spectra for the original base liquors revealed that, while their overall spectral shapes were similar, significant differences existed in finer details. All three base liquors exhibited a prominent fluorescence peak at a wavelength of 432 nm. This shared peak indicates that the Baijiu base liquors samples belong to the same flavor type and brand, suggesting similar overall chemical compositions. However, subtle differences arise due to trace compounds, such as esters and acids, which undergo reactions such as esterification, redox transformations, and decomposition over time, influencing the detailed fluorescence characteristics of Baijiu base liquors.

For instance, the fluorescence peak of the 1-year-aged base liquor appeared at a wavelength of 310 nm, while the peak for the 5-year-aged base liquor shifted to 344 nm. This shift indicates the evolution of chemical components during aging. Additionally, the fluorescence intensity of Baijiu base liquors increased with storage duration, reflecting changes in its chemical composition. The aging process not only altered the position of fluorescence peaks but also enhanced overall fluorescence intensity, offering insights into the gradual transformations occurring in Baijiu base liquors over time.

**Fig 5:**
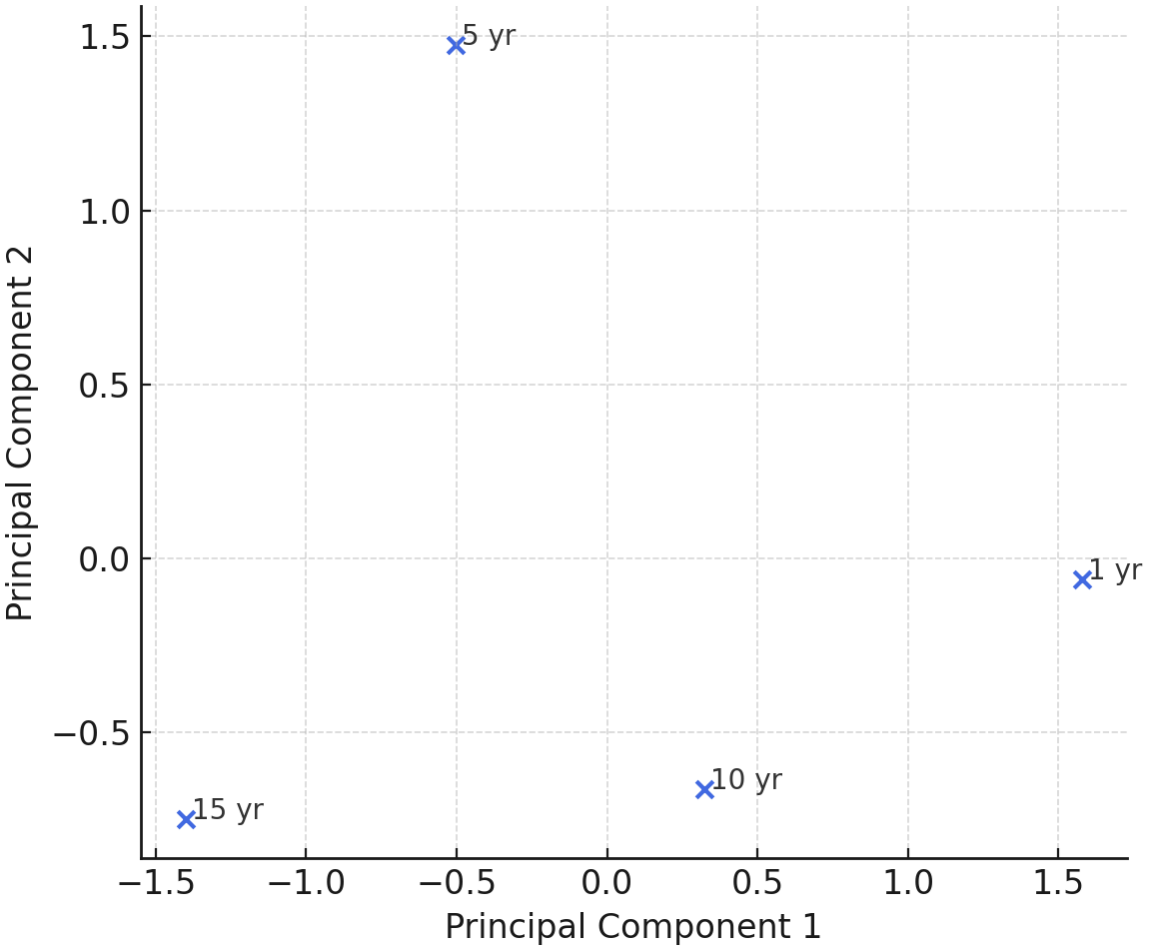
Principal component Analysis (PCA) scores of fluorescence spectra of Baijiu base liquor with different aging periods.

**Fig 6:**
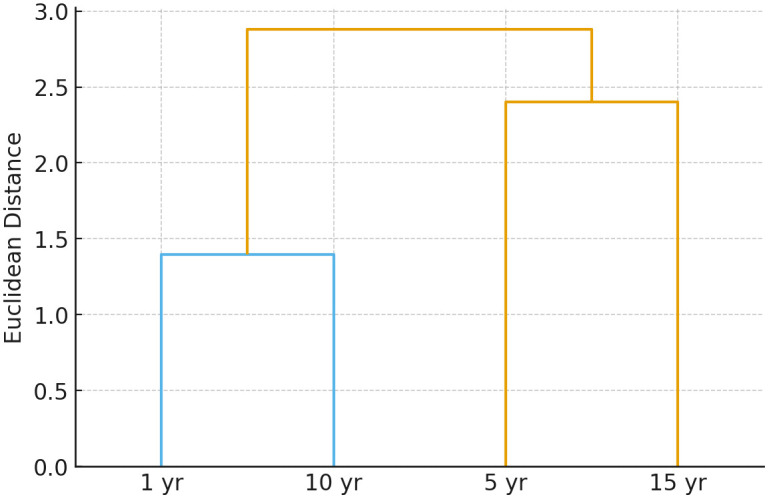
Hierarchical cluster analysis (HCA) dendrogram of fluorescence spectral data of Baijiu base liquor with different aging periods.

When these base liquors were blended into aged Baijiu base liquors, the resulting fluorescence spectra retained the main characteristics of the original base liquors. The blended aged Baijiu base liquors exhibited fluorescence peaks near 340 nm, 434 nm, and 538 nm. These peaks highlight the combined characteristics of the blending process and the original base liquors. To accurately distinguish various aged Baijiu base liquor types based on fluorescence characteristics, principal component analysis (PCA) and hierarchical cluster analysis (HCA) were applied to the fluorescence spectral data. The PCA score plot (Fig. 5) showed clear separation among samples from 1-, 5-, 10-, and 15-year aging groups, indicating distinct fluorescence evolution patterns over time. Similarly, the HCA dendrogram (Fig 6) clustered the samples according to their aging sequence, confirming the strong correlation between fluorescence features and storage duration.

## 4. Discussion

This study systematically revealed the compositional changes of Luzhou-flavor Baijiu base liquors base liquors across different aging periods by combining mass spectrometry and three-dimensional fluorescence spectroscopy. The results demonstrated that key components such as esters and acids undergo significant concentration changes during aging. Moreover, a “redshift” phenomenon was observed in the fluorescence spectra of base liquors as aging time increased, reflecting the gradual molecular structural complexity and enriched flavor layers. These findings provide critical evidence for the complexification mechanisms of Baijiu base liquors flavors during aging and propose an approach to optimize flavor by controlling changes in fluorescence spectral characteristics, offering theoretical support and technical methods for Baijiu base liquors quality improvement.

In analyzing the chemical compositional changes during Baijiu base liquors aging, this study referenced numerous foundational studies, which provided methodological inspiration and findings, though with limitations. This study innovatively expanded upon those foundations. Chen et al. [23] employed headspace gas chromatography-ion mobility spectrometry (HS-GC-IMS) to investigate the volatile components of Baijiu base liquors during aging and their changes but did not delve into the dynamic relationships among multiple components. Building on the integration of mass spectrometry and fluorescence spectroscopy, this study quantified the concentrations and spectral changes of various chemical components, achieving a comprehensive analysis of the dynamic relationships among components and revealing how interactions between esters and acids influence the overall flavor of the liquor during aging. Yan et al. [24] utilized high-performance liquid chromatography combined with fluorescence spectroscopy to explore the oxidative polymerization phenomena of polyphenols in Baijiu base liquors base liquors. However, their study did not analyze the synergistic effects of polyphenols with other key components. This study further refined the analysis of fluorescence changes in polyphenolic components and uncovered the mechanisms by which these, in conjunction with other components, contribute to the “redshift” phenomenon. This provided more precise data supporting the contribution of polyphenols to the overall flavor. Wang et al. [19] applied two-dimensional gas chromatography-time-of-flight mass spectrometry (GC × GC-TOFMS) to monitor the fluctuations of ester and acid components during specific stages of Baijiu base liquors aging. However, their research primarily focused on the changing characteristics of components at different aging stages without deeply analyzing their relative stability.The samples aged under standardized constant temperature and humidity conditions exhibited higher stability of flavor components, suggesting that strict environmental control contributes to maintaining aroma consistency. Fluorescence spectroscopy was then introduced for dynamic monitoring of component fluctuations during aging, evaluating the stability of base liquor components and providing new insights into maintaining Baijiu base liquors flavor consistency.

To further explain the observed relationship between the decline in ester concentrations and the accumulation of acids, this study hypothesizes that the underlying mechanism may be driven by acid-catalyzed hydrolysis reactions. During the aging process—particularly under long-term storage conditions—ester compounds are prone to acid-catalyzed hydrolysis, yielding the corresponding organic acids and alcohols. For instance, ethyl hexanoate can undergo hydrolysis in an acidic environment to produce hexanoic acid and ethanol. The former tends to accumulate over time during aging, which aligns with the increasing acid concentration observed in this study. Additionally, elevated storage temperature and prolonged aging duration may further facilitate such hydrolytic reactions, providing a plausible explanation for the year-by-year decrease in ester levels alongside the continuous rise in acid content. Similarly, the increasing concentration of polyphenolic compounds may result from the oxidative polymerization of phenolic substances under oxidative conditions, forming high-molecular-weight conjugated systems that enhance fluorescence intensity. This phenomenon corresponds with the observed “redshift” in fluorescence spectra. By integrating these proposed transformation pathways, this study constructs a conceptual conversion network of key flavor compounds during the aging process of Baijiu base liquor, offering a theoretical foundation for future mechanistic validation and flavor modulation strategies.

The contributions of this study include the innovative use of mass spectrometry and three-dimensional fluorescence spectroscopy, which not only enriched the methodological system for studying Baijiu base liquors base liquor compositional changes but also provided higher precision detection tools for the field. Additionally, this research proposed a novel approach to flavor optimization by monitoring fluorescence spectral characteristics, offering a new technical pathway for Baijiu base liquors quality enhancement with practical significance. However, some limitations remain. The research samples were exclusively drawn from Luzhou-flavor Baijiu base liquors produced in Sichuan Province. As a result, the observed patterns and mechanisms may not be directly generalizable to all types of Chinese Baijiu, particularly those with different stylistic profiles such as sauce-aroma or rice-aroma types. This may limit the broader applicability of the findings. Furthermore, although this study employed mass spectrometry for the quantitative analysis of key compounds and utilized fluorescence spectroscopy to reveal spectral evolution characteristics of flavor substances, the application of these techniques primarily focused on changes in compound concentrations and spectral responses. There was limited exploration of inter-molecular reaction mechanisms—such as esterification, oxidation, or polymerization—and a systematic reaction mechanism model has yet to be established.

## 5. Conclusion

This study systematically analyzed the chemical evolution of Luzhou-flavor Baijiu base liquors from Sichuan Province across different aging periods using mass spectrometry and three-dimensional fluorescence spectroscopy. The main conclusions are as follows:

(1) The heatmap results revealed that ester compounds peaked at 95.56 mg/L during the 5-year aging period, followed by a significant decline to 24.04 mg/L by year 20. Acid compounds increased to a maximum concentration of 97.29 mg/L at year 5 and then gradually decreased. Alcohols and polyphenols reached their respective peaks at year 10 (87.96 mg/L) and year 15 (80.67 mg/L), after which their concentrations also declined. These findings demonstrate a staged evolution pattern among various flavor compounds, highlighting the dynamic transformation characteristics of key components during the Baijiu aging process
(2) OPLS-DA analysis revealed significant separation of samples from different aging periods in the principal component space. One-year-aged base liquor was dominated by ester compounds, characterized by a prominent fruity aroma. In 10-year-aged samples, the combined effects of esters and acids led to a more balanced flavor profile. By 20 years, acids became the dominant component, significantly enhancing the overall flavor complexity. The dynamic changes of key compounds further elucidate the dominant trends in chemical transformations during the aging process.
(3) Three-dimensional fluorescence spectroscopy revealed that the fluorescence peak of the base liquor shifted from 420 nm to 480 nm during aging. This “redshift” phenomenon reflects the oxidative polymerization of polyphenolic compounds, which enhances the complexity and depth of Baijiu base liquors ’s flavor.

The study uncovered the chemical evolution patterns of Luzhou-flavor Baijiu base liquors across different aging periods, providing scientific evidence for improving Baijiu base liquors quality and optimizing the aging process. Future research could expand the sample scope to include base liquors of different regions and flavor types, enhancing the generalizability of the conclusions. In addition, the integration of advanced analytical techniques and data-driven methods can further deepen the investigation. On one hand, techniques such as nuclear magnetic resonance (NMR) and high-resolution mass spectrometry (HRMS) can be employed to elucidate the structural changes and reaction pathways of key flavor compounds during aging. When combined with stable isotope labeling, these tools allow for precise tracking of the formation and transformation of target compounds, thereby enabling the construction of empirically supported reaction network models. On the other hand, machine learning algorithms can be applied to extract features and develop predictive models based on multidimensional spectral and mass spectrometric data, facilitating intelligent identification of aging stages and flavor evolution trends. This approach supports smart development in Baijiu base liquors quality monitoring, process optimization, and precision flavor control. Moreover, the fluorescence “redshift” feature proposed in this study shows considerable potential for industrial applications in flavor evolution monitoring. In future work, this characteristic signal could be integrated into dynamic monitoring systems through online fluorescence sensor technologies, enabling real-time assessment of flavor compounds and early quality warnings, thereby advancing the intelligence level of Baijiu production processes.

## Supporting information

S1 Fig Figure2-Data. Score plot of OPLS-DA for different aging periods (1 year, 10 years, 20 years) of Baijiu base liquors.
(XLSX)

S2 Fig Figure3-Data. Odor activity value (OAV) contributions of key compounds for different aging periods (1 year, 10 years, 20 years) of Baijiu base liquors.
(XLSX)

S3 Fig Figure4-Data. The 3D fluorescence spectra of the original base liquors with ages of (a) 1 years, (b) 5 years and (c) 10 years, as well as the blended aged liquors with weighted ages of (d) 5 years, (e) 10 years and (f) 15 years.
(XLSX)

S1 Table Supplementary Data for Table 1. Table 1. List of markers.
(XLSX)

S2 Table Supplementary Data for Table 2. Table 2. Categories of Flavor Compounds in Baijiu Base Liquors and Their Flavor Contributions.
(XLSX)

S3 File Example calculations. (DOCX)

S1 File Supporting information. (XLSX)
