# Supplementary material for: Research on the aging characteristics of base liquor in Sichuan Province’s strong Aroma Baijiu at different aging periods using mass spectrometry and 3D fluorescence spectroscopy methods
Source: PLoS One. 2026 Apr 30;21(4):e0344656. doi: 10.1371/journal.pone.0344656 (PMC13132190; doi:10.1371/journal.pone.0344656)
Supplement: S3 File — (DOCX) [file pone.0344656.s006.docx]

**Supplementary . Example calculation of equivalent aging time**

To demonstrate the practical applicability of the effective aging model, example calculations of the equivalent aging time T were performed using the blending schemes described in Section 2.1 of the main text.

The effective aging degree of a base liquor aged for time t is defined as:

$$M(t)=1-e^{-kt}$$

where k=0.12 year^−1^.

For a blended liquor composed of multiple base liquors, the equivalent aging time T is calculated as:

$$T=-\frac{1}{k}\ln\left(\sum_{i} w_{i}e^{-kt_{i}}\right)$$

Example 1: Blended liquor with an equivalent aging time of 5 years

According to the blending scheme described in the manuscript, the 5-year blended sample consisted of 70% 1-year base liquor and 30% 10-year base liquor. Thus, w_1_=0.7, t_1_=1 year; w_2_=0.3, t_2_=10 years.

$$\begin{matrix} \sum_{i} w_{i}e^{-kt_{i}} & =0.7e^{-0.12\times1}+0.3e^{-0.12\times10} \\ & =0.7\times0.887+0.3\times0.301=0.711 \\ T & =-\frac{1}{0.12}\ln(0.711)\approx4.9 \text{years} \end{matrix}$$

The calculated equivalent aging time is close to the nominal value of 5 years.
